# Therapeutic Drug Monitoring and Pharmacogenomics of Thiopurines in Inflammatory Bowel Disease: International Guidelines Revisited

**DOI:** 10.1097/FTD.0000000000001373

**Published:** 2025-08-29

**Authors:** Ahmed B. Bayoumy, Luc J. J. Derijks, Nanne K. H. de Boer

**Affiliations:** * Department of Gastroenterology and Hepatology, Amsterdam University Medical Center, Amsterdam, the Netherlands;; † Amsterdam Gastroenterology Endocrinology Metabolism Research Institute, Amsterdam, the Netherlands;; ‡ Department of Clinical Pharmacy and Pharmacology, Máxima Medical Center, Veldhoven, the Netherlands; and; § Department of Clinical Pharmacy and Toxicology, Maastricht University Medical Center, and NUTRIM, School of Nutrition and Translational Research in Metabolism, Maastricht University, Maastricht, the Netherlands.

**Keywords:** thiopurines, inflammatory bowel disease, 6-thioguanine nucleotides, thiopurine methyltransferase, NUDT15

## Abstract

**Background:**

Thiopurines, including azathioprine, mercaptopurine, and thioguanine (TG), are widely used as maintenance therapies for inflammatory bowel disease (IBD). However, their clinical utility is challenged by interindividual variability in metabolism, therapeutic response, and toxicity. Therapeutic drug monitoring (TDM) and pharmacogenomics have emerged as critical tools for optimizing thiopurine therapies. This review aimed to compare the international guidelines on TDM and the pharmacogenomics of thiopurines in IBD to identify global differences in TDM practices.

**Methods:**

A scoping review was conducted using PubMed, Embase, Web of Science, and official Gastroenterology Society web sites. We identified and analyzed international guidelines for thiopurine TDM and pharmacogenomics, focusing on recommendations for metabolite monitoring, thiopurine methyltransferase, and nudix hydrolase 15 (NUDT15) testing.

**Results:**

A total of 23 guidelines from North America, Europe, Africa, Asia, Latin America, and Oceania were included in this study. TDM was recommended in 65% of the guidelines with various approaches; some advocated reactive monitoring (6-TGN and/or 6-MMPR in response to treatment failure or adverse effects), whereas others recommended proactive thiopurine monitoring to prevent toxicity. Thiopurine methyltransferase testing is recommended in 74% of these guidelines, although its relevance has been questioned in Asian populations. NUDT15 testing is mentioned in only 13% of the guidelines, despite its established role in predicting thiopurine-induced myelotoxicity, particularly in Asian and Hispanic populations. Accessibility limitations in low-resource regions have led to a reliance on empirical dose adjustments and complete blood count monitoring.

**Conclusions:**

There is substantial variability in the global TDM and pharmacogenomic practices for thiopurines in IBD. Limited recommendations for NUDT15 testing and disparities in resource availability have contributed to inconsistent clinical implementation. Future guidelines should address these gaps by integrating cost-effective pharmacogenomic strategies and incorporating alternative monitoring methods such as DNA-TG for NUDT15 variants. This review highlights the variability in the global recommendations for thiopurine TDM and pharmacogenomics. To optimize thiopurine therapy and reduce toxicity risks, future guidelines should include NUDT15 testing and consider TG, low-dose thiopurine, and allopurinol treatments.

## Abstract 2

Supplemental Digital Content is Available in the Text.

## INTRODUCTION

Thiopurines, including azathioprine (AZA), mercaptopurine (MP), and thioguanine (TG), are widely used as maintenance therapies in the management of inflammatory bowel disease (IBD). Despite their efficacy, thiopurines present significant challenges related to interindividual variability in metabolism, therapeutic response, and adverse effects.^1^ Important side effects include the occurrence of myelotoxicity and hepatotoxicity. Therapeutic drug monitoring (TDM) and pharmacogenomics have emerged as critical tools for optimizing thiopurine therapy and ensuring efficacy while minimizing toxicity.^2^ National and regional guidelines have been developed to provide recommendations for the implementation of TDM and pharmacogenomic testing for thiopurines in IBD. These guidelines aim to standardize clinical practice by offering evidence-based recommendations for dosing, monitoring strategies, and genetic testing of thiopurine methyltransferase (TPMT) and nudix hydrolase 15 (NUDT15) variants. TPMT variants are more common among European and African ancestries, whereas NUDT15 variants are more common among Asian and Hispanic ancestries.^3,4^ For TDM, 6-thioguanine nucleotides (6-TGN) and/or 6-methylmercaptopurine ribosides (6-MMPR) can be used to monitor therapy.^5^ High 6-TGN and 6-MMPR levels are related to bone marrow and liver toxicity, whereas low 6-TGN levels are associated with treatment failure and nonadherence.^2,6^ This is particularly useful in patients with wild-type metabolism and TPMT variants, but less useful for NUDT15 variants.^7^ By examining these variations, we aim to provide insights into optimizing thiopurine therapy and identify potential areas for harmonization in clinical practice. This review aimed to compare international guidelines on TDM and the pharmacogenomics of thiopurines in IBD to identify global differences in TDM practices.

## METHODS

### Data Sources and Search Strategy

A literature search was conducted using the PubMed, Embase, and Web of Science electronic databases. In addition, guidelines were retrieved from international therapeutic drug monitoring and pharmacogenomics guidelines, the official web sites of leading gastroenterology and hepatology societies, including the American Gastroenterological Association (AGA), European Crohn's and Colitis Organisation (ECCO), Asian Organization for Crohn's and Colitis (AOCC), Latin American Society for Crohn's and Colitis (SLCC), and Australian and New Zealand IBD Guidelines. The search queries are provided in **Supplemental Digital Content 1**, http://links.lww.com/TDM/A882.

### Data Extraction

In this scoping review, we focused on statements regarding thiopurine metabolite monitoring, TPMT, and/or NUDT15 testing. If reported, we included the percentage of consensus in the individual guidelines or consensus statements. We also included evidence and/or recommendation grading if reported. The first author, country, region, and year of publication were included in the data extraction process. **Supplemental Digital Content 2**, http://links.lww.com/TDM/A882, provides the full text of the recommendations in the guidelines.

### Methodology for Guideline Appraisal

**FIGURE 1.**
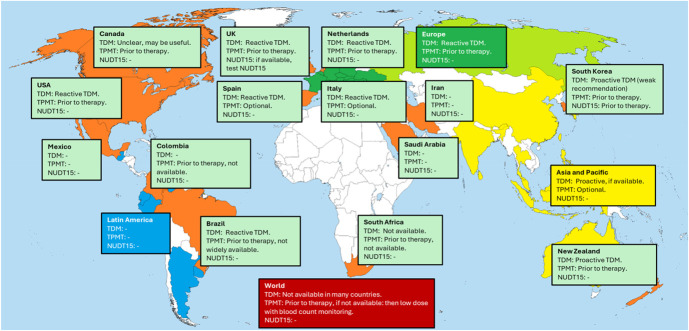
Overview of global thiopurine TDM and pharmacogenomics guidelines. Orange: countries with individual guidelines (light green boxes). Yellow (Asia and Pacific)/blue (Latin America)/green (Europe): regional guidelines. Bright green: Russia (banned from ECCO). Red: World Gastroenterology Organization.

The quality of the clinical guidelines was assessed using the Appraisal of Guidelines for Research and Evaluation II (AGREE II) instrument. This validated tool evaluates the methodological rigor and transparency with which the guidelines were developed. The AGREE II comprises 23 items organized into 6 domains: (1) Scope and Purpose, (2) Stakeholder Involvement, (3) Rigor of Development, (4) Clarity of Presentation, (5) Applicability, and (6) Editorial Independence. Each item was scored on a 7-point Likert scale (1 = strongly disagree, 7 = strongly agree) by 2 independent reviewers (A.B. and L.D.R.). Domain scores were calculated by summing the individual item scores within each domain and scaling the total score as a percentage of the maximum possible score. Discrepancies between the reviewers were resolved through discussion or consultation with a third reviewer (N.K.H.d.B). The results were used to inform the overall assessment of the guideline quality and recommendations for use (Fig. 1 and Table 1).^8–31^

**TABLE 1.** Recommendations Regarding Thiopurine Metabolite Monitoring and Pharmacogenomics in Thiopurine-Treated Patients With IBD

Author	Country	Year	Thiopurine Metabolite Monitoring	TPMT Genotyping	NUDT15 Genotyping	Evidence and Recommendations
Feuerstein et al.^8^	USA	2017	Favors: Reactive thiopurine metabolite monitoring Against: Routine monitoring of thiopurine metabolites Additional: 6-TGN cut-off 230 and 450 pmol/8 × 10^8^ RBC	Favors: Routine TPMT testing at the start of therapy to guide thiopurine dosing Additional: Routine laboratory monitoring, including complete blood count, is recommended	Not mentioned	Thiopurine metabolite monitoring *Conditional recommendation, very low quality of evidence* TPMT testing *Conditional recommendation, low quality* **AGREE II: 7**
Bressler et al.^9^	Canada	2015	Favors: Unclear; thiopurine metabolite levels may be helpful to guide therapy	Favors: TPMT testing before therapy to identify patients at risk for myelosuppression Additional: TPMT testing does not replace monitoring of CBC.	Not mentioned	GRADE: Weak recommendation, low-quality evidence; vote: strongly agree, 22%; agree, 78% **AGREE II: 7**
Bernstein et al.^10^	Global	2015	Issue: Thiopurine metabolite monitoring is not available in many countries (not specifically mentioned which countries)	TPMT available: No AZA or MP if TPMT function is deficient, lower thiopurine dosage if TPMT activity is below normal TPMT not available: thiopurine dose should be escalated from 50 mg to the full dose while monitoring the blood count	Not mentioned	Not mentioned **AGREE II: 3**
ECCO e-Guide 2.0^11^	Europe	2019	Favors: Reactive thiopurine monitoring 6-TGN cut-off levels not mentioned	Low TPMT level: 50% of the thiopurine dose Absent TPMT level: Do not use thiopurines Additional: TPMT testing cannot substitute for complete blood count monitoring	Not mentioned	Not mentioned **AGREE II: 5**
Lamb et al.^12^	UK	2019	Favors: Reactive thiopurine monitoring 6-TGN high: Restart at a lower dose once the abnormality has resolved, and monitor hematology and thiopurine metabolites 6-MMP high: Consider restarting low-dose thiopurine with allopurinol 100 mg	Favors: Routine TPMT testing at the start of therapy Normal TPMT: 2 mg/kg AZA or 1 mg/kg MP. Low TPMT activity: Avoid thiopurines Intermediate TPMT activity: 50% of thiopurine dose; 1 mg/kg AZA or 0.5 mg/kg MP.	If available, test NUDT15 genotype	Thiopurine metabolite monitoring (GRADE: weak recommendation, low-quality evidence; agreement: 81.4%) TPMT testing (GRADE: strong recommendation, moderate-quality evidence; agreement: 100%) **AGREE II: 7**
Bermejo et al.^13^	Spain	2018	Thiopurine metabolite testing: Poor treatment adherence, underdosed patients, hepatotoxicity, or myelotoxicity Hypermethylation (hepatotoxicity/lack of response): Low doses of thiopurines and allopurinol, monitored by metabolite levels	TPMT activity assays (phenotyping or genotyping) are not essential for starting thiopurine therapy; if such assays are possible, they will allow greater initial safety	Not mentioned	Not mentioned **AGREE II: 4**
Biancone et al.^14^	Italy	2017	Favors: Reactive thiopurine monitoring 6-Thioguanine levels have been associated with thiopurine efficacy, whereas 6-MMP levels are associated with drug-related toxicity	Optional: Testing for TPMT activity has been suggested to predict thiopurine toxicity; the cost-effectiveness of TPMT testing needs to be re-evaluated	Not mentioned	Not mentioned **AGREE II: 4**
Relling et al.^15^	Global (CPIC ®)	2018	Erythrocyte TGNs or MeMPNs are not related to NUDT15 genotypes, but there is evidence that intermediate and poor metabolizers for NUDT15 accumulate higher levels of DNA-TG than normal metabolizers	Dose adjustments based on disease-specific guidelines	Dose adjustments based on disease-specific guidelines	Different recommendations are given for different TPMT/NUDT15 phenotypes; see article by Relling et al.^15^ **AGREE II: 6**
Van Bodegraven et al.^16^	The Netherlands	2015	Favors: Reactive monitoring (in case of incompliance, efficacy, or toxicity) *Routine use of thiopurine metabolites is not recommended* In thiopurine-failing patients who have aberrant thiopurine metabolism, the addition of allopurinol (100 mg) combined with dose-reduced thiopurine (25%–33% of the original dose) is a successful treatment **AZA/MP** Underdosing/noncompliance 6-TGN <250, 6-MMPR <5700 Therapeutic level: 6-TGN 250–500, 6-MMPR <5700 Potential myelotoxicity (absent TPMT activity) 6-TGN >> 500, 6-MMPR << 5700 Possible myelotoxicity (low TPMT activity) 6-TGN >500, 6-MMPR <5700 Possible hepatotoxicity (high TPMT activity) 6-TGN <250–500, 6-MMPR >5700 Potential myelotoxicity (very high TPMT activity): 6-TGN << 250, 6-MMPR >> 5700 6-TGNs according to the Lennard method^17^ *The guideline mentions that different metabolite levels for TG can be found in a supplement file, but does not provide this file*	TPMT testing before therapy, followed by dose adjustments, leads to safer therapy without loss of efficacy	Not mentioned	Not mentioned **AGREE II: 7**
Derijks et al.^18^	The Netherlands	2018	Therapeutic 6-TGN levels AZA/MP: 230–450 (Lennard method^17^) TG: 230–1000 (Lennard method^17^) Toxic 6-TGN levels AZA/MP: >450 (6-TGN, Lennard method^17^), >5700 (6-MMP, Lennard method^17^) TG: >2000 (6-TGN, Lennard method^17^)	TPMT testing before therapy AZA/MP TPMT EM: 100% standard dose TPMT IM: 50% standard dose TPMT PM: 0%–10% standard dose TG TPMT EM: 100% standard dose TPMT IM: 75% standard dose TPMT PM: 10% standard dose	Not mentioned	Not mentioned **AGREE II: 5**
Lee et al.^19^	South Korea	2015	The dose adjustment of thiopurines through monitoring of 6-TGN and 6-MMP levels is expected to improve efficacy and reduce side effects in patients treated with thiopurines	Assessment of the TPMT genotype or enzyme activity before initiating thiopurine therapy is of limited value in east-Asian populations compared with that in Caucasians	NUDT15 genotyping before thiopurine therapy may identify patients at risk of thiopurine-induced early leukopenia	Thiopurine metabolite monitoring Quality of evidence: Moderate Classification of recommendation: Weak Level of agreement: Strongly agree 18%, agree 69%, uncertain 13% TPMT testing Quality of evidence: Moderate Classification of recommendation: Weak Level of agreement: Strongly agree 29%, agree 55%, uncertain 16% NUDT15 testing Quality of evidence: Moderate Classification of recommendation: Strong Level of agreement: Strongly agree 13%, agree 76%, uncertain 11% **AGREE II: 5**
Ran et al.^20^	Asia-Pacific	2021	Not mentioned	Rejected statements regarding TPMT/NUDT15 testing	Rejected statements regarding TPMT/NUDT15 testing	Not mentioned **AGREE II: 3**
Ooi et al.^21^	Asia-Pacific	2015	Not mentioned	Evidence of the benefit of TPMT testing in Asia is required before its routine use can be recommended	Not mentioned	TPMT/NUDT15 testing Level of agreement: a—74%, b—26%, c—0%, d—0%, e—0% Quality of evidence: III Classification of recommendation: C **AGREE II: 3**
Ooi et al.^22^	Asia-Pacific	2010	Thiopurine metabolite testing for 6-TGN and 6-MMP may assist dose optimization to avoid drug-induced toxicity; lower starting doses in Asian compared with White populations, along with close monitoring of CBC and liver function, are recommended	Optional: Where available, TPMT may assist dose optimization of AZA/6-MP to avoid drug-induced toxicity	Not mentioned	Not specifically mentioned for thiopurine metabolite or TPMT testing **AGREE II: 4**
Khan et al.^23^	New Zealand	2019	Indiscernible or negligible 6TGN and 6MMP: Medication nonadherence (check adherence) Low 6-TGN and low 6-MMP: Underdosing or medication nonadherence (increase dose and monitor after checking adherence) Low 6-TGN and high 6-MMP (6-TGN:6-MMP>20): Thiopurine hypermethylator or “shunter” (add allopurinol and reduce thiopurine dose to 25%–33%). Normal 6TGN* (235–450) and 6MMP <5700 mol/8 × 10^8^ RBC: Adequate dosing, continue therapy or escalate therapy if active IBD High 6-TGN and high 6-MMP: Increased risk of adverse events (decrease dose; add another drug if active disease) 6-TGNs according to the Lennard method^17^	Favors: TPMT enzyme activity testing is recommended in all patients commencing thiopurine Treatment initiation with 33%–50% of the target dose is recommended in patients with intermediate TPMT enzyme activity and at 10% of the target dose in patients with low or absent enzyme activity	Not mentioned	Not mentioned **AGREE II: 6**
Mosli et al.^24^	Saudi Arabia	2022	Not mentioned	Not mentioned	Not mentioned	Not mentioned **AGREE II: 3**
Khoshnam-Rad et al.^25^	Iran	2023	Not mentioned	Not mentioned	Not mentioned	Not mentioned **AGREE II: 3**
Watermeyer et al.^26^	South Africa	2021	Monitoring of thiopurine metabolites is not available in South Africa	TPMT testing should be considered before the initial use of AZA or MP	Not mentioned	Not mentioned **AGREE II: 4**
Imbrizi et al.^27^	Brazil	2023	Low-dose thiopurines (25%–33% of the usual dose) in combination with allopurinol 100 mg might be considered in patients with thiopurine hepatotoxicity, nausea, or flu-like symptoms, or those who are hypermethylators	Thiopurines are avoided in low TPMT activity; dose reduction of 50% in intermediate TPMT activity; TPMT genotyping/phenotyping is not widely available in Brazil	Not mentioned	Not specifically mentioned for thiopurine metabolite or TPMT testing **AGREE II: 4**
Juliao-Baños et al.^28^	Colombia	2020	Not mentioned	TPMT testing should be considered before starting thiopurines, if available The TPMT test is not available in Colombia	Not mentioned	Not mentioned **AGREE II: 4**
Yamamoto et al.^29^	Mexico (CD)	2024	Not mentioned	Not mentioned	Not mentioned	Not mentioned **AGREE II: 3**
Yamamoto et al.^30^	Mexico (UC)	2018	Not mentioned	Not mentioned	Not mentioned	Not mentioned **AGREE II: 3**
Yamamoto et al.^31^	Latin America	2017	Not mentioned	Not mentioned	Not mentioned	Not mentioned **AGREE II: 3**

CBC, complete blood count; EM, extensive metabolizer; IM, intermediate metabolizer; PM, poor metabolizer.

## RESULTS

This review included 22 international and regional guidelines on thiopurine TDM and pharmacogenomics for IBD. These guidelines have been published by leading gastroenterology societies and 2 pharmacology/pharmacogenomic groups across North America, Europe, Africa, Asia, Latin America, and Oceania. The guidelines include varied recommendations regarding TDM implementation, metabolite thresholds, and pharmacogenomic testing strategies.

### Therapeutic Drug Monitoring

Recommendations for thiopurine metabolite monitoring were mentioned in 15 (65%) guidelines. Five guidelines advocate the use of reactive thiopurine metabolite monitoring (ie, using 6-TGNs and/or 6-MMPR in case of effectiveness and/or side effects).^8,11–14,16,27^ Other guidelines take a more proactive approach where thiopurine metabolites are measured to prevent side effects (eg, early detection of shunters) or to assess underdosing.^9,15,18,19,22,23^ Guidelines have also mentioned the use of low dose AZA/MP and allopurinol in patients with thiopurine hypermethylation.^12,23,27^ Specific thiopurine metabolite cut-off levels have been mentioned in only 3 guidelines.^8,18,23^ Lee et al^19^ mentioned that dose adjustments through 6-TGN and 6-MMPR monitoring may improve efficacy and reduce side effects, without providing details of what action should be taken at which specific thiopurine metabolite levels. A similar statement was made by Ooi et al,^22^ who additionally mentioned that thiopurine dosage should be started at a lower level in Asians than in Whites. Specific 6-TGN and 6-MMP levels were mentioned by Khan et al,^23^ van Bodegraven et al,^16^ and Derijks et al.^18^ All guidelines use the Lennard method^17^ to measuring thiopurine metabolites. However, the cutoff values between the guidelines were slightly different. Khan et al^23^ use 6-TGN levels of 235–450 pmol/8 × 10^8^ RBC as the normal range, similar to Derijks et al^18^ who used 230–450 pmol/8 × 10^8^ RBC. Van Bodegraven et al^16^ use a slightly higher cutoff value of 250–500 pmol/8 × 10^8^ RBC. All guidelines use a cut-off value for 6-MMPR of 5700 pmol/8 × 10^8^ RBC. Although recommendations regarding the use of thiopurine metabolites have been mentioned in European, North American, and Asian guidelines, these recommendations differ in other parts of the world. In the African, South American, and World Gastroenterology Organization (WGO) guidelines, thiopurine metabolite testing is not widely available in these countries; therefore, no recommendations have been made. The Clinical Pharmacogenetics Implementation Consortium (CPIC) guidelines state that RBC 6-TGNs are not useful for NUDT15 genotypes, and there is evidence that DNA-incorporated TG (DNA-TGs) are related to NUDT15 variants.^15^

### TPMT Testing

Recommendations regarding TPMTs were mentioned in 17 (74%) guidelines. Most guidelines recommend TPMT before initiating thiopurine therapy. Dose reductions based on TPMT are mentioned in 5 guidelines.^11,12,18,23,27^ Some guidelines also mention that TPMT does not replace complete blood count monitoring (CBC).^8,9,11^ Guidelines from Spain and Italy do not strongly recommend the routine use of TPMT. Notably, guidelines from Asian countries mention the limited applicability of TPMT assessment in Asian populations compared with White populations.^19,21^ Furthermore, guidelines from South Africa,^26^ Brazil,^27^ Colombia,^28^ and the WGO^10^ mentioned that although TPMT testing is recommended, it is not widely available. Specifically, the WGO guideline^10^ mentioned that if TPMT testing is not available, patients should be considered to start using thiopurines at low doses with escalating dosages until the full dose is achieved using close monitoring of CBC.^10^

### NUDT15 Testing

The recommendation for NUDT15 testing was only mentioned in 3 (13%) guidelines.^12,15,19^ In the Korean guidelines by Lee et al,^19^ NUDT15 genotyping has been recommended before starting thiopurine therapy to prevent early leukopenia. In the BSG guidelines, NUDT15 testing is recommended if the test is available.^12^ Furthermore, it was mentioned that the recommendation for TPMT/NUDT15 testing has been rejected by the consensus workgroup in Asia and the Pacific. It was mentioned in the consensus statement that NUDT15 is probably more relevant in Asian patients than TPMT.^20^ None of the IBD guidelines mentioned any advice regarding dose adjustments in NUDT15 variant patients.^12,19^ Recommendations regarding dosing were mentioned in the CPIC guidelines for both malignant and nonmalignant conditions.^15^

## DISCUSSION

This review assessed 23 international thiopurine TDM and pharmacogenomic guidelines for patients with IBD. Our findings highlight the substantial variability in recommendations across different regions, particularly in the implementation of thiopurine metabolite monitoring, TPMT, and NUDT15 testing. Although thiopurines are increasingly being supplanted by biologics in some (mostly high-income) parts of the world, guidelines are needed to optimally manage patients with IBD treated with thiopurines.^32^

### Pharmacogenomics

Future efforts should focus on harmonizing global recommendations and improving access to cost-effective pharmacogenomic testing. To encourage broader adoption of TPMT and NUDT15 testing, especially in regions where these tests are currently unavailable, sequencing technologies must become more affordable and accessible.^33,34^ TPMT is among the most extensively studied genetic polymorphisms.^35^ This is probably the reason why 74% of the guidelines mention it. Nevertheless, concrete (dosing) advice was mostly lacking (mentioned in 5 studies), even though this was well studied in the pivotal TOPIC trial.^36^ NUDT15 is one of the essential issues that should be addressed in future guidelines, especially considering that NUDT15 variants are associated with a higher risk of developing myelotoxicity in IBD patients.^37^ These NUDT15 variants are common among Asian and Hispanic populations but are also present in multiethnic groups in the United States and Europe.^4,38,39^ Special attention should also be given to patients with both a TPMT and NUDT15 variant. Until now, none of the IBD-specific guidelines have focused on these patients. Furthermore, most guidelines were published 5–15 years ago, and it is possible that the latest data on NUDT15 variants were unknown at that time.

The major issue for TDM in patients with NUDT15 variant is that 6-TGN measurements are less useful for monitoring because 6-TGN comprised the sum of the individual 6-thioguanine monophosphate, diphosphate, and triphosphate metabolites and NUDT-15 affects the interrelationship between these 3 components.^40^ DNA-TG predicts late leukopenia in patients with IBD and NUDT15 variants, whereas 6-TGNs were not able to predict leukopenia in patients with IBD and NUDT15 variants.^41^ Future studies should also consider using DNA-TG as a method for monitoring NUDT15 variant patients and should investigate its clinical utility in guiding dose adjustments.^7,15^ If validated, DNA-TG could be integrated into future guidelines as an alternative or complementary biomarker for TDM in patients with NUDT15 variants and possibly the broader population of patients (without variants of thiopurine metabolism) treated with thiopurines. However, DNA-TG requires LC-MS/MS and may be less available because of the cost of investment in resource-limited settings.

### Therapeutic Drug Monitoring

TDM is recommended in 63% of the IBD guidelines, which outline various practices and recommendations. Most guidelines that include thiopurine metabolite measurements suggest reactive TDM. Some guidelines do not specify the exact 6-TGN and/or 6-MMP cut-off levels. In addition, the guidelines do not specifically mention which method or assay was used to measure thiopurine metabolites. Although cut-off levels for thiopurines are well-established for AZA/MP, the cut-off levels for TG remain less defined.^42,43^ Regarding the cost-effectiveness of thiopurine metabolite monitoring, a Singaporean study found that monitoring 6-TGN and 6-MMP levels was able to identify shunters (n = 9), subtherapeutic (n = 28), and supratherapeutic (n = 19) 6-TGN levels, without significantly increasing overall costs.^44^ Dubinsky et al^45^ found that thiopurine metabolite monitoring (6-TGN <235 pmol/8 × 10^8^ RBC) also led to reduced total costs (incremental costs: -$701) with improved therapy outcome (3.75 weeks earlier response). These findings suggest that monitoring thiopurine metabolites is a cost-effective strategy.

### Innovative Treatments in Aberrant Thiopurine Metabolism

The use of low-dose AZA/MP and allopurinol has not been widely mentioned in guidelines but has been recognized in various studies as an alternative treatment option in case of thiopurine hypermethylation.^46,47^ Another option would be to consider TG in future guidelines as a maintenance therapy option in case of failure (eg, side effects or hypermethylation) of AZA/MP. The advantage of TG is that it can be directly converted to 6-TGNs without the formation of toxic 6-MMPR.^48,49^

### Addressing Global Disparities

Our findings highlighted significant disparities in the implementation of TDM and pharmacogenomic testing across different guidelines. Although TPMT and, to a lesser extent, NUDT15 testing have been incorporated into guidelines in high-income countries, resource-limited regions rely more on empirical dose adjustments and CBC monitoring, whereas precisely in these latter regions thiopurines are and will continue to be an important treatment option. During CBC monitoring, the use of mean corporal volume (MCV) has been considered in some reports for monitoring patients treated with thiopurines in resource-limited settings. MCV was found to be a surrogate marker to predict 6-TGN levels in some studies, although 6-TGNs are superior.^50^ Although this strategy can mitigate early toxicity, it does not fully eliminate the risk of severe adverse reactions in patients with high-risk TPMT or NUDT15 variants. Different strategies can be implemented in pharmacogenomics and TDM depending on the setting and local context. Furthermore, collaborating with (pediatric) oncology centers could be an additional option to further increase the number of tests and justify investments in thiopurine pharmacogenomic testing in settings where these tests do not yet exist.

Another critical step in reducing disparities is lowering the costs for pharmacogenetic testing.^33^ Next-generation sequencing and point-of-care genetic testing have the potential to make TPMT and NUDT15 screening more accessible, even in regions with limited laboratory infrastructure. Pharmacogenomic testing (TPMT) for AZA was found to be cost-effective in 7 of the 9 studies that evaluated its cost-effectiveness.^51^ In the long run, these strategies may even be more cost-effective for IBD patients treated with thiopurines in lower- and middle-income countries; however, these cost-effectiveness studies should also be performed in these settings. Governmental and nongovernmental organizations should subsidize these initiatives to ensure that patients in economically disadvantaged regions who rely on these immunomodulators are not excluded from cost-effective personalized medicine approaches.

## CONCLUSIONS

This review underscores the variability in the global recommendations for TDM and pharmacogenomics in IBD. Although some regions have adopted TDM and pharmacogenetic screening, others face limitations in access and feasibility. NUDT15 testing should be included in future guidelines, and the use of TG, low-dose thiopurine, and allopurinol should be considered for hypermethylation of AZA/MP.

## Supplementary Material
