# Supplementary material for: Therapeutic Drug Monitoring and Pharmacogenomics of Thiopurines in Inflammatory Bowel Disease: International Guidelines Revisited
Source: Ther Drug Monit. 2025 Aug 29;48(3):400–8. doi: 10.1097/FTD.0000000000001373 (PMC13152070; doi:10.1097/FTD.0000000000001373)
Supplement: Supplementary file 1 [file tdm-48-400-s001.docx]

**Supplement S1. Search queries for different guidelines.**

**PubMed Search Query**

**("Inflammatory Bowel Diseases"[MeSH] OR "IBD" OR "Crohn Disease"[MeSH] OR "Ulcerative Colitis"[MeSH] OR "Crohn’s disease" OR "ulcerative colitis")**

**AND ("Thiopurines"[MeSH] OR "Azathioprine"[MeSH] OR "6-Mercaptopurine"[MeSH] OR "6-TG" OR "6-MP" OR "Thiopurine metabolites")**

**AND ("Guideline"[Publication Type] OR "Consensus Statement" OR "Practice Guidelines" OR "International Guidelines" OR "Clinical Guidelines")**

**Embase Search Query**

**('inflammatory bowel disease'/exp OR 'ibd' OR 'crohn disease'/exp OR 'ulcerative colitis'/exp OR 'crohn’s disease' OR 'ulcerative colitis')**

**AND ('thiopurine'/exp OR 'azathioprine'/exp OR '6-mercaptopurine'/exp OR '6-thioguanine' OR 'thiopurine metabolites')**

**AND ('guideline'/exp OR 'consensus statement' OR 'practice guideline' OR 'international guideline' OR 'clinical guideline')**

**Web of Science**

**TS=("Inflammatory Bowel Disease" OR "IBD" OR "Crohn's Disease" OR "Ulcerative Colitis")**

**AND TS=("Thiopurine" OR "Azathioprine" OR "6-Mercaptopurine" OR "6-TG" OR "6-MP" OR "Thiopurine Metabolites")**

**AND TS=("Guideline" OR "Consensus Statement" OR "Practice Guideline" OR "International Guideline" OR "Clinical Guideline")**

**Supplement S2. The full text of the guidelines on TDM and pharmacogenomics of thiopurines.**

| **Author** | **Thiopurine metabolite monitoring** | **TPMT genotyping** | **NUDT15 genotyping** |
| --- | --- | --- | --- |
| Feuerstein et al.^8^ | *In adult patients treated with thiopurines with active IBD or adverse effects thought to result from thiopurine toxicity, the AGA suggests reactive thiopurine metabolite monitoring to guide treatment changes. Conditional recommendation, very low quality of evidence.*  *When measuring thiopurine metabolite monitoring in patients with active IBD-related symptoms, we suggest a target 6-thioguanine (6-TGN) cut-off between 230 and 450 pmol/8 × 10^8^ red blood cells (RBCs) when used as monotherapy; the optimal 6-TGN cut-off when thiopurines are used in combination with anti-TNF agents is uncertain.*  *In adult patients with quiescent IBD treated with thiopurines, the AGA suggests against routine thiopurine metabolite monitoring. Conditional recommendation, very low quality of evidence.* | *In adult patients with IBD being started on thiopurines, the AGA suggests routine TPMT testing (enzymatic activity or genotype) to guide thiopurine dosing.*  *Conditional recommendation, low quality*  *Routine laboratory monitoring, including CBC, should be performed, regardless of TPMT testing results.* | *Not mentioned.* |
| Bressler et al.^20^ | *In addition, higher levels of the thiopurine metabolite 6-thioguanine nucleotide have been correlated with clinical remission rates; therefore, thiopurine metabolite levels may be helpful to guide therapy* | *The TPMT assay is necessary before initiation of treatment to identify patients at risk for severe dose-dependent myelosuppression.*  *TPMT testing does not replace the need for mandatory monitoring of complete blood cell count.* | *Not mentioned.* |
| Bernstein et al.^25^ | *Thiopurine metabolite tests are not available in many countries, but where available can help explain the lack of response.* | *Do not offer azathioprine or mercaptopurine for CD or UC if thiopurine methyltransferase activity (TPMT) is deficient. Use at a lower dose if TPMT activity is below normal.*  *If TPMT measurement is not available, the thiopurine dose should be escalated from 50 mg to the full dose while monitoring the blood count. Asians appear to require lower doses of thiopurine to achieve efficacy, and the full dosage is usually limited by the development of cytopenia.*  *Patients should be monitored for neutropenia if they are taking azathioprine or mercaptopurine, even if TPMT enzyme levels are normal.* | *Not mentioned.* |
| ECCO e-Guide 2.0^9^ | *If patients relapse on therapy, optimize dose: Metabolite testing (6-TGN and MMP).* | *Reduce the dose by 50% if the TPMT level is low, but do not use thiopurines if TPMT is very low/absent (increased risk of developing severe, life-threatening myelotoxicity). TPMT testing cannot substitute for complete blood count (CBC) monitoring in patients receiving thiopurines.* | *Not mentioned.* |
| Lamb et al.^10^ | *Thiopurine metabolites (TGN and MeMP) can be used to optimize drug dosing. We suggest that metabolite monitoring may be used for those with inadequate response to therapy or toxicity, but should not be a substitute for routine monitoring of blood tests (GRADE: weak recommendation, low-quality evidence. Agreement: 92.9%). We suggest that low-dose thiopurines (25–33% of the usual dose) used in combination with allopurinol 100 mg may be considered in patients with thiopurine hepatotoxicity, nausea or flu-like symptoms, or those who are hypermethylators (GRADE: weak recommendation, low-quality evidence. Agreement: 81.4%).*  *Check thiopurine metabolites and alter dosage.*   - *If TGN is high, then restart at a lower dose once the abnormality has resolved and monitor hematology and thiopurine metabolites carefully* - *If MeMP is high, then consider restarting low-dose thiopurine with allopurinol 100 mg* - *If TGN is low or normal, then likely to recur. Advise to stop thiopurine in this circumstance* | *We recommend that all IBD patients considered for thiopurine therapy should have an assessment of thiopurine methyltransferase (TPMT) status (GRADE: strong recommendation, moderate-quality evidence. Agreement: 100%).*  *We recommend that thiopurines should be avoided in patients with low TPMT activity. The dose of thiopurine should be reduced to 50% in those with intermediate thiopurine activity. (GRADE: strong recommendation, moderate-quality evidence. Agreement: 100%).*  *Check TPMT and start at the target dose once the result is available. Normal TPMT: 2 mg/kg azathioprine or 1 mg/kg mercaptopurine. Low: 1 mg/kg azathioprine or 0.5 mg/kg mercaptopurine. Very low: avoid thiopurine.* | *If available, test NUDT15 genotype.* |
| Bermejo et al.^11^ | *It is possible to determine blood 6-TGN and 6-MMP concentration, which allows subjects with hypermethylation to be identified and underdosing and lack of treatment adherence to be detected.* | *TPMT activity assays are not essential for starting thiopurine therapy. Nevertheless, if such assays are possible, they will allow greater safety at the start of treatment.* | *Not mentioned.* |
| Biancone et al.^12^ | *6-Thioguanine levels have been associated with thiopurine efficacy, while 6-MMP levels are associated with drug-related toxicity.* | *Testing for TPMT activity has therefore been suggested to predict thiopurine toxicity, including bone marrow suppression, although with conflicting findings. The cost-effectiveness of TPMT testing needs to be re-evaluated* | *Not mentioned.* |
| Relling et al.^19^ | Erythrocyte TGNs or MeMPNs are not related to NUDT15 genotypes, but there is evidence that intermediate and poor metabolizers for NUDT15 accumulate higher levels of DNA-TG than normal metabolizers. | Dose adjustments based on disease-specific guidelines. | Dose adjustments based on disease-specific guidelines. |
| Van Bodegraven et al.^14^ | Favors: reactive monitoring (in case of incompliance, efficacy, or toxicity).  *Routine use of thiopurine metabolites is not recommended.*  In thiopurine-failing patients who have aberrant thiopurine metabolism, the addition of allopurinol (100 mg) combined with dose-reduced thiopurine (25-33% of the original dose) is a successful treatment.  **AZA/MP**  Underdosing/non-compliance 6-TGN < 250, 6-MMPR <5700  Therapeutic level: 6-TGN 250-500, 6-MMPR < 5700  Potential myelotoxicity (absent TPMT activity) 6-TGN >> 500, 6-MMPR << 5700  Possible myelotoxicity (low TPMT activity) 6-TGN > 500, 6-MMPR < 5700  Possible hepatotoxicity (high TPMT activity) 6-TGN <250-500, 6-MMPR > 5700  Potential myelotoxicity (very high TPMT activity): 6-TGN << 250, 6-MMPR >> 5700.  6-TGNs according to the Lennard method.^21^  *The guideline mentions that different metabolite levels for TG can be found in a supplement file, but the guideline does not provide this supplement file.* | TPMT testing before therapy, followed by dose adjustments, leads to safer therapy without loss of efficacy. | Not mentioned. |
| Derijks et al.^18^ | Therapeutic 6-TGN levels  AZA/MP: 230-450 (Lennard method^21^)  TG: 230-1000 (Lennard method^21^)  Toxic 6-TGN levels  AZA/MP: >450 (6-TGN, Lennard method^21^), > 5700 (6-MMP, Lennard method^21^).  TG: >2000 (6-TGN, Lennard method^21^). | TPMT testing before therapy.  AZA/MP  TPMT EM: 100% standard dose  TPMT IM: 50% standard dose  TPMT PM: 0-10% standard dose  TG  TPMT EM: 100% standard dose  TPMT IM: 75% standard dose  TPMT PM: 10% standard dose | *Not mentioned.* |
| Lee et al.^17^ | *The Dose Adjustment of Thiopurines Through Monitoring of 6-TGN and 6-Methylmercaptopurine (6-MMP) Levels Is Expected to Improve Efficacy and Reduce Side Effects in Patients Treated With Thiopurines. Quality of evidence: Moderate Classification of recommendation: Weak Level of agreement: Strongly agree 18%, Agree 69%, Uncertain 13%* | *Assessment of the TPMT Genotype or Enzyme Activity Before Initiating Thiopurine Therapy Is of Limited Value in East-Asian Populations Compared to That in Caucasians.*  *Quality of evidence: Moderate*  *Classification of recommendation: Weak*  *Level of agreement: Strongly agree 29%, Agree 55%, Uncertain 16%* | *Determination of NUDT15 Genotypes Before*  *Initiating Thiopurine Therapy May Identify Patients With a Predisposition to the Development of Thiopurine-Induced Early Leukopenia.*  *Quality of evidence: Moderate*  *Classification of recommendation: Strong*  *Level of agreement: Strongly agree 13%, Agree 76%, Uncertain 11%* |
| Ran et al.^26^ | Not mentioned. | *Eight statements were rejected as this indicated that consensus had not been reached. The statement “IBD patients should receive TPMT and NUDT15 polymorphism test before thiopurine treatment” was rejected.* | *In Asian patients, it is strongly recommended to detect the NUDT15 genotype rather than TPMT before initiating thiopurine therapy.* |
| Ooi et al.^22^ | Not mentioned. | *Patients on thiopurines need close monitoring for myelotoxicity and hepatotoxicity. More evidence of the benefit of thiopurine methyl transferase (TPMT) testing in Asia is required before its routine use can be recommended.*  *Level of agreement: a—74%, b—26%, c—0%, d—0%, e—0%*  *Quality of evidence: III*  *Classification of recommendation: C* | Not mentioned. |
| Ooi et al.^16^ | *Thiopurine metabolite testing for 6-thioguanine and 6-methylmercaptopurine may assist dose optimization of AZA/6-MP to avoid drug-induced toxicity.*  *Lower starting doses in Asian compared to Caucasian populations, along with close monitoring of complete blood count and liver function, are recommended.* | *Where available, TPMT may assist dose optimization of AZA/6-MP to avoid drug-induced toxicity.* | Not mentioned. |
| Khan et al.^15^ | *In 90% of patients with normal TPMT*  *activity, target doses will usually be in the*  *order of 2–3mg/kg for AZA and 1–1.5mg/kg*  *for MP, with dose adjustment based on thiopurine metabolite concentrations.*   - *Indiscernible or negligible 6TGN and 6MMP: medication non-adherence (check adherence).* - *Low 6-TGN and low 6-MMP: Underdosing or medication non-adherence (increase dose and monitor after checking adherence).* - *Low 6-TGN and high 6-MMP (6-TGN:6-MMP>20): Thiopurine hypermethylator or “shunter” (add allopurinol and reduce thiopurine dose to 25-33%).* - *Normal 6TGN* (235-450) and 6MMP<5700 pmol/8×10^8^ RBC: adequate dosing, continue therapy or escalate therapy if active IBD.* - *High 6-TGN and high 6-MMP: increased risk of adverse events (decrease dose. Add another drug if active disease).* | *Testing for TPMT enzyme activity is recommended in all patients commencing a thiopurine.*  *Treatment initiation with 33–50% of the*  *target dose is recommended in patients with intermediate TPMT enzyme activity, and at 10% of the target dose in patients with low or absent enzyme activity* | Not mentioned. |
| Mosli et al.^47^ | Not mentioned. | Not mentioned. | Not mentioned. |
| Khoshnam-Rad et al.^48^ | Not mentioned. | Not mentioned. | Not mentioned. |
| Watermeyer et al.^23^ | *Monitoring of thiopurine metabolites is not available in South Africa.* | *TPMT testing should be considered before the initial use of AZA or MP to treat patients with IBD.* | Not mentioned. |
| Imbrizi et al.^13^ | *Low-dose thiopurines (25-33% of the usual dose) in combination with allopurinol 100 mg might be considered in patients with thiopurine hepatotoxicity, nausea, or flu-like symptoms, or those who are hyper-methylators.* | *Thiopurines should also be avoided in patients with low TPMT activity. The dose of thiopurine should be reduced to 50% in those with intermediate TPMT activity.*  *In the Brazilian public health scenario, analytical methods for the measurement of TPMT genotype and phenotypic activity are not widely available.* | Not mentioned. |
| Juliao-Baños et al.^24^ | Not mentioned. | *TPMT enzyme activity can be measured before starting the administration of thiopurines, since this allows the identification of patients who may develop severe immunosuppression if these drugs are used. Currently, the TPMT test is not available in Colombia; however, starting its use in the country would be an ideal scenario since patients treated with thiopurines may develop immunosuppression. The future inclusion of this test in the health benefits plan of the mandatory health insurance coverage system in force in Colombia is suggested.* | Not mentioned. |
| Yamamoto et al.^49^ | Not mentioned. | Not mentioned. | Not mentioned. |
| Yamamoto et al.^50^ | Not mentioned. | Not mentioned. | Not mentioned. |
| Yamamoto et al.^51^ | Not mentioned. | Not mentioned. | Not mentioned. |
